# Facile Synthesis, Characterization, and Photocatalytic Evaluation of In_2_O_3_/SnO_2_ Microsphere Photocatalyst for Efficient Degradation of Rhodamine B

**DOI:** 10.3390/nano12183151

**Published:** 2022-09-11

**Authors:** Chunlan Wang, Gangying Guo, Changjun Zhu, Yuqing Li, Yebo Jin, Bingsuo Zou, Han He, Aolin Wang

**Affiliations:** 1 School of Science, Xi’an Polytechnic University, Xi’an 710048, China; 2 Guangxi Key Laboratory of Processing for Nonferrous Metals and Featured Material, School of Resources, Environment and Materials, Guangxi University, Nanning 530004, China

**Keywords:** In_2_O_3_/SnO_2_, photocatalysis, Rhodamine B, electron transfer

## Abstract

The tin dioxide (SnO_2_) photocatalyst has a broad application prospect in the degradation of toxic organic pollutants. In this study, micron-sized spherical SnO_2_ and flower indium oxide (In_2_O_3_) structures were prepared by a simple hydrothermal method, and the In_2_O_3_/SnO_2_ composite samples were prepared by a “two-step method”. Using Rhodamine B (RhB) as a model organic pollutant, the photocatalytic performance of the In_2_O_3_/SnO_2_ composites was studied. The photocurrent density of 1.0 wt.% In_2_O_3_/SnO_2_ was twice that of pure SnO_2_ or In_2_O_3_, and the degradation rate was as high as 97% after 240 min irradiation (87% after 120 min irradiation). The reaction rate was five times that of SnO_2_ and nine times that of In_2_O_3_. Combined with the trapping experiment, the transient photocurrent response, and the corresponding characterization of active substances, the possible degradation mechanism was that the addition of In_2_O_3_ inhibited the efficiency of electron–hole pair recombination, accelerated the electron transfer and enhanced the photocatalytic activity.

## 1. Introduction

The rapid development of modern industry has led to a large number of new organic pollutants that are difficult to degrade into water bodies, which pose a significant potential threat to human, animal and environmental health [1]. Therefore, there is an urgent need for economic and efficient catalysts and technologies to degrade organic pollutants [2]. It is worth noting that the textile industry discharges the largest amount of dye wastewater, accounting for almost half of the organic pollutant wastewater worldwide [3]. Textile dyes are also classified as carcinogenic and neurotoxic, causing respiratory infections, skin, gastrointestinal irritation and eye infections, with developmental and mimic toxicity in animals and humans [4]. Therefore, it is still a great challenge to find an efficient method for handling industrial wastewater.

The catalysts are the “chip” of wastewater treatment, which have always been closely related to the development and application of the modern chemical industry, and are also emerging as a potential material to promote energy conversion and storage applications [5,6]. In recent years, advanced oxidation processes (AOPs) have received more and more attention as an effective method to decompose organic wastewater [7]. At the same time, researchers emphasize that the varying chemical and structural properties of catalysts should be the focus when analyzing the active factors of catalysts [8]. It is well known that various AOPs have been used for the decontamination of wastewater, in which photocatalytic degradation is one of the most fascinating techniques owing to its simplicity, superior removal efficiency and lack of secondary pollution.

Rhodamine B (RhB) is one of the most toxic dyes in textile wastewater. Due to its high stability and non-biodegradability, it is often chosen as a typical model dye for photocatalytic degradation experiments. Many scientists tried to degrade RhB with different semiconductor photocatalysts, such as TiO_2_ [9], SnO_2_ [10], ZnO [11], WO_3_ [12] and In_2_O_3_ [13]. Among all the photocatalysts, due to its advantages of environmental friendliness, non-toxicity, low cost, excellent photoelectrochemical properties, and especially its lower conduction band (CB) position, SnO_2_ is a better electron acceptor than TiO_2_ and ZnO [14]. However, due to the high recombination rate of photogenerated electron–hole pairs in pure SnO_2_, the photocatalytic efficiency is low. Therefore, it is crucial to promote the photocatalytic performance of SnO_2_ by accelerating the separation of photoinduced charge pairs. In particular, the preparation of composites by coupling SnO_2_ with other photocatalytic materials has been shown to be an effective strategy for suppressing the rapid complexation of photogenerated electron–hole pairs [15]. Furthermore, the photocatalytic activity of SnO_2_ is significantly enhanced when the metal oxide is coupled with short-bandgap or wide-bandgap semiconductors [16]. Indium oxide (In_2_O_3_) is also an important metal–oxide semiconductor, which has been proved to be an effective sensitizer. Due to its perfect physicochemical properties, aqueous stability and low toxicity, it is a suitable candidate for the effective photodegradation of pollutants in wastewater [17]. Therefore, the coupling effect of SnO_2_ and In_2_O_3_ was chosen to enhance the photocatalytic activity.

In this study, micron-sized spherical SnO_2_ and flower In_2_O_3_ structures were prepared by a simple hydrothermal method. In_2_O_3_-doped SnO_2_ (In_2_O_3_/SnO_2_) samples with different percentages of In_2_O_3_ contents were prepared by a “two-step method”. The crystal structure and physical properties of the In_2_O_3_/SnO_2_ composites were studied in detail. Using RhB as a model organic pollutant, the photocatalytic performance of the In_2_O_3_/SnO_2_ composites was studied. Finally, the photodegradation mechanism of the In_2_O_3_/SnO_2_ composites was discussed.

## 2. Materials and Methods

### 2.1. Materials

All the chemicals used in this study were purchased from Sinopharm Chemical Reagent Co., Ltd. (Shanghai, China), and were of analytical grade purity and did not require further purification. All the solutions were prepared with deionized (DI) water.

### 2.2. Synthesis of Photocatalysts

SnO_2_ was prepared by the hydrothermal method [18]. Firstly, 1.4 g SnCl_4_·5H_2_O, 2.94 g Na_3_C_6_H_5_O_7_·2H_2_O and 0.16 g NaOH were dissolved in a mixture of 30 mL absolute ethanol and 45 mL deionized (DI) water, and then stirred vigorously for 1 h to form a homogeneous solution. Then, it was transferred to a Teflon-lined stainless-steel autoclave and heated at 180 °C for 12 h. After cooling to room temperature, the precipitate was collected by centrifugation, then washed successively with deionized water and absolute ethanol. Finally, the obtained SnO_2_ was dried at 60 °C in air for 12 h.

In_2_O_3_ was also prepared by a simple hydrothermal route [19]. Firstly, 0.42 g InCl_3_·4H_2_O and 1.22 g sodium dodecyl sulfate (SDS) were dispersed into 80 mL deionized water and stirred for 30 min. Next, 0.42 g CO(NH_2_)_2_ was added to the above mixed solution and stirred vigorously for 1 h. The solution was completely dissolved and transferred to a stainless-steel autoclave lined with Teflon, placed in an electric oven and heated at 120 °C for 12 h. The following steps were the same as synthesizing the SnO_2_. Finally, the synthesized samples were calcined in a Muffle furnace at 500 °C for 2 h at a heating rate of 2 °C/min. After cooling to room temperature, In_2_O_3_ was obtained.

In_2_O_3_/SnO_2_ was prepared by the hydrothermal method. Firstly, 30 mL absolute ethanol and 45 mL DI water were mixed and stirred for 30 min to form solution A. Different amounts of In_2_O_3_ were dispersed into solution A and ultrasonically dispersed for 30 min to form solution B. Next, 1.4 g SnCl_4_·5H_2_O was added to solution B and fully dissolved. Then, 2.94 g Na_3_C_6_H_5_O_7_·2H_2_O was added and stirred for 20 min until completely dissolved. Finally, 0.16 g NaOH was added and stirred for 10 min to form solution C. The following steps were the same as the synthesis of SnO_2_. The synthesized sample was calcined in Muffle furnace at 700 °C for 1 h. After cooling to room temperature, the composite material In_2_O_3_/SnO_2_ was synthesized. The amounts of In_2_O_3_ were 0.1, 0.5, 1.0 and 1.5 wt.%.

### 2.3. Characterization of Photocatalysts

The crystal structure of the composite was studied by Mini Flex600 X-ray diffractometry (XRD, Rigaku, Tokyo, Japan, Cu-Kα, 10°~80°, 5° min^−1^). A field emission scanning electron microscope (SEM, Oxford Anta-450, FEI, Oxford, England) characterized the morphology of the synthetic materials. Transmission electron microscopy (TEM, Tecnai G2, FEI, Oxford, England) was used to obtain the morphology and element distribution. The surface chemical composition was investigated by X-ray photoelectron spectroscopy (XPS, Thermo ESCALAB 250X, Waltham, MA, USA, C1s revised at 284.8 eV). The absorption spectra were carried out by a UV–Visible spectrophotometer (Lambda 750, Perkin-Elmer, Shelton, CT, USA) and the electron–hole binding capacity was examined by photoluminescence spectroscopy (PL, HORIBA FluoroMax-4, Piscataway, NJ, USA) using an excitation wavelength of 320 nm.

### 2.4. Photocatalytic Activity Tests

The photocatalytic performances of the catalyst were investigated by the photodegradation of RhB under the ultraviolet light irradiation of a 200 W Hg lamp (CEL-LAM500, Beijing Au Light, Beijing, China). In all the experiments, 50 mg of the catalyst powder was dispersed into 50 mL of the RhB solution (10 mg·L^−1^). Before the photoreaction began, the solution was magnetically mixed in the dark for 30 min to complete the adsorption–desorption equilibrium. During the photoreaction, 1.6 mL of the suspension was collected every 20 min and centrifuged. The strongest absorption peak of the RhB solution was determined by a UV–Vis–NIR PE Lambda 950 spectrophotometer. In the cycle experiment, the samples were washed with DI and absolute ethanol several times before starting the new cycle photocatalytic test. In addition, triethanolamine (TEA) was used as hole radical, p-benzoquinone (BQ) as a superoxide radical, and tert-butanol (TBA) as a hydroxyl radical.

### 2.5. Photocurrent Experiments

The photochemical properties of the samples were studied by a photocurrent experiment. The prepared samples were coated onto FTO glass as the working electrode, platinum wire was used as the counter electrode, Ag/AgCl as the counter reference electrode, and 0.1 M Na_2_SO_4_ as the electrolyte solution. A bias potential of 0.5 V was applied to the photoanode when the photocurrent test was performed under switching light conditions. Electrochemical impedance spectroscopy (EIS) measurements were carried out in the frequency range of 0.01 Hz–10 kHz in 0.1 M Na_2_SO_4_ solution, and were performed using ZENNIUM electrochemical workstation (Zahner Instruments, Kronach, Germany). The electrochemical signals for all the tests were recorded by a CHI660E electrochemical analyzer (Chenhua Instruments, Shanghai, China).

## 3. Results and Discussion

### 3.1. Morphology and Structure of the Catalyst

**Figure 1:**
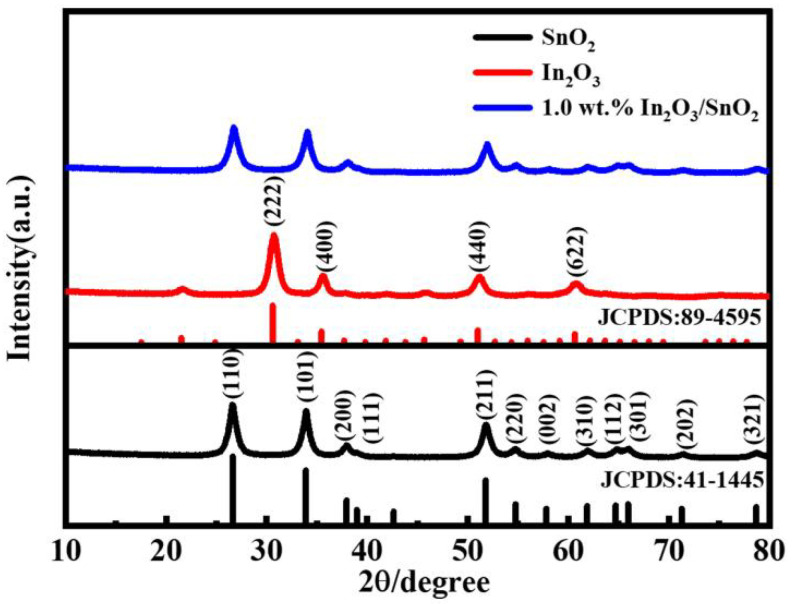
XRD patterns of pure SnO_2_, In_2_O_3_ and 1.0 wt.% In_2_O_3_/SnO_2_ composite photocatalysts.

The crystalline phases of the synthesized materials were investigated using the XRD method, and the effects are shown in Figure 1. The significant characteristic peaks of pure SnO_2_ at 2θ = 26.6°, 33.9°, 37.9°, 51.8°, 54.8° and 65.9° were attributed to the (110), (101), (200), (211), (220) and (301) crystal planes of tetragonal SnO_2_ (JCPDS No.41-1445), respectively [20]. The distinctive characteristic peaks of In_2_O_3_ at 2θ = 30.6°, 35.5°, 51.0° and 60.7° were attributed to the (222), (400), (440) and (622) crystal planes of cubic In_2_O_3_ (JCPDS No.89-4595), respectively [19]. The SnO_2_ peak intensity of the 1.0 wt.% In_2_O_3_/SnO_2_ photocatalyst was lower than that of pure SnO_2_. The study determined that the doping of In_2_O_3_ did not appreciably alter the crystal shape of SnO_2_. This may have also been due to the low-doping-content material of In_2_O_3_ in the composites. Additionally, it was shown that “In” was integrated into the SnO_2_ lattice.

**Figure 2:**
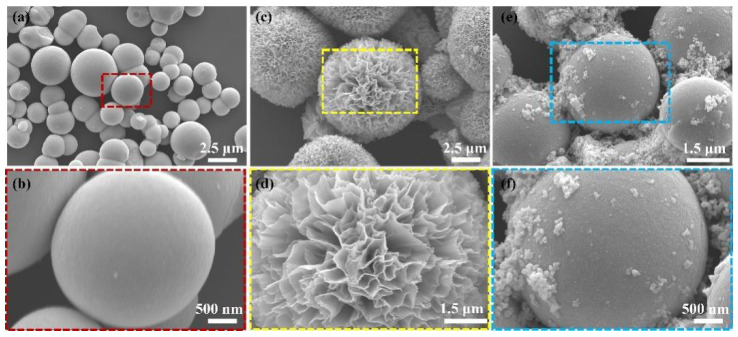
SEM images: (**a**,**b**) of the pure SnO_2_; (**c**,**d**) of the prepared In_2_O_3_; (**e**,**f**) of the prepared 1.0 wt.% In_2_O_3_/SnO_2_ composite photocatalysts.

The morphology and structure of the synthesized materials were investigated by SEM. The effects are shown in Figure 2, and Figure 2b,d,f show the magnifications of Figure 2a,c,e, respectively. Figure 2a,b show that SnO_2_ was a spherical structure in which the particle size was about 2.8 μm. Figure 2c,d show that In_2_O_3_ was a three-dimensional flower-like structure consisting of an effective stacking of nanosheets, of which the particle size was about 8.3 μm. Figure 2e,f show the morphological structure of the 1.0 wt.% In_2_O_3_/SnO_2_ photocatalyst, and a layer of In_2_O_3_ attached to the surface of SnO_2_.

**Figure 3:**
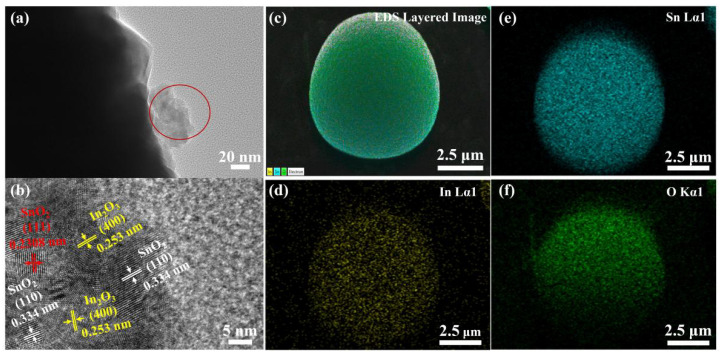
TEM image: (**a**) of the prepared 1.0 wt.% In_2_O_3_/SnO_2_ composite photocatalysts; (**b**) HRTEM images; TEM–EDS elemental mapping of 1.0 wt.% In_2_O_3_/SnO_2_ composite photocatalysts: (**c**) EDS layered image; (**d**) In Lα1; (**e**) Sn Lα1; (**f**) O Kα1.

TEM and HRTEM analysis was performed on the 1.0 wt.% In_2_O_3_/SnO_2_ composite photocatalyst material. Figure 3a is the TEM image of the 1.0 wt.% In_2_O_3_/SnO_2_ photocatalyst. It was found that not only was a layer of In_2_O_3_ attached to the surface of SnO_2_, but a portion of In_2_O_3_ was also embedded in the SnO_2_ sphere. Figure 3b shows the HRTEM image of the selected part of Figure 3a. The fringes with the surface spacing of 0.2308 nm and 0.334 nm belonged to the lattice surface of SnO_2_, corresponding to the spacings of (111) and (110) of SnO_2_ (JPCDS 41-1445), respectively. The fringe with a surface spacing of 0.253 nm belonged to the lattice surface of In_2_O_3_, corresponding to the (400) spacing of In_2_O_3_ (JCPDS No.89-200 4595). The crystallinity and composition of the samples were analyzed by TEM–EDS. The elemental composition and distribution of the 1.0 wt.% In_2_O_3_/SnO_2_ samples were studied by TEM–EDS spectroscopy (Figure 3c–f). In the 1.0 wt.% In_2_O_3_/SnO_2_ sample, the elements of Sn, In and O were uniformly distributed. These results confirmed the formation of the In_2_O_3_/SnO_2_ heterostructure, which was beneficial for suppressing electron–hole recombination.

**Figure 4:**
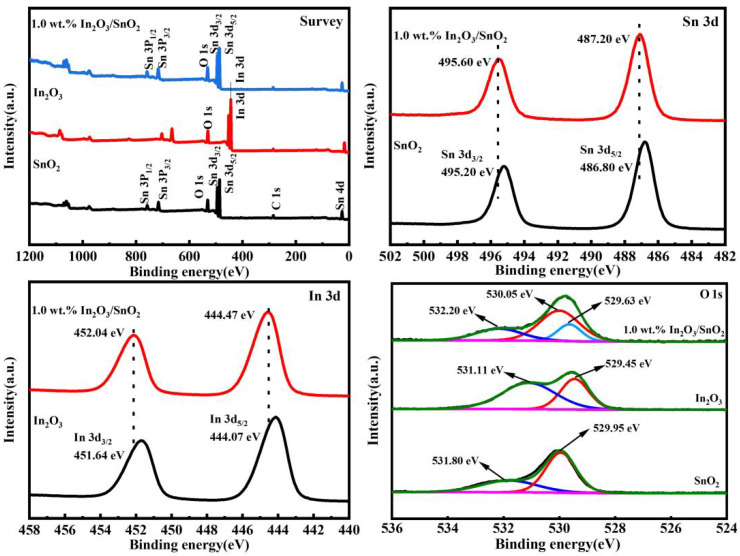
XPS spectra of pure SnO_2_, In_2_O_3_ and 1.0 wt.% In_2_O_3_/SnO_2_ composite photocatalysts: (**a**) survey; (**b**) Sn3d; (**c**) In3d; (**d**) O1s.

The chemical state and surface composition of the synthesized materials were investigated by XPS. As shown in Figure 4a, the XPS survey spectrum showed characteristic peaks of Sn, In, O and C elements. The spectrum was calibrated relative to the C-element peak. Figure 4b shows the Sn3d spectrum of SnO_2_ with two peaks at 486.80 eV and 495.20 eV for the Sn3d_5/2_ and Sn3d_3/2_ orbitals of Sn^4+^, respectively [21]. In addition, after binding with In_2_O_3_, the binding energies of Sn3d_5/2_ and Sn3d_3/2_ of the 1.0 wt.% In_2_O_3_/SnO_2_ photocatalyst shifted to 487.20 eV and 495.60 eV, respectively.

Figure 4c shows that the In3d spectrum of In_2_O_3_ has two peaks at 440.07 eV and 451.64 eV, belonging to the In3d_5/2_ and In3d_3/2_ orbitals of In^3+^, respectively [22]. In addition, the binding energies of the 1.0 wt.%In_2_O_3_/SnO_2_ photocatalysts for In3d_5/2_ and In3d_3/2_ were shifted to 444.47 eV and 452.04 eV, respectively. The characteristic peaks of Sn3d and In3d for the 1.0 wt.% In_2_O_3_/SnO_2_ photocatalyst had a negative shift of 0.4 eV. This was due to the strong electronic interaction between SnO_2_ and In_2_O_3_, was beneficial to improve the migration efficiency of photogenerated carriers at the interface [23].

Figure 4d shows the XPS spectra of In_2_O_3_, SnO_2_ and the 1.0 wt.% In_2_O_3_/SnO_2_ photocatalyst O1s. The asymmetric O1s in SnO_2_ was decomposed into two peaks located around 529.95 eV and 531.80 eV. One of the bands placed at 531.80 eV (O_v_) belonged to the O atoms close to OVs or hydroxyl-like groups. Another band at 529.95 eV (O_latt_) was viewed to be the Sn-O-Sn oxygen bond. The asymmetric O1s in In_2_O_3_ was decomposed into two peaks. One of the bands at 531.11 eV (O_v_) belonged to the O atoms close to OVs or hydroxyl-like groups. Another band at 529.45 eV (O_latt_) was considered to be the In-O-In oxygen bond. The O1s spectrum of the 1.0 wt.% In_2_O_3_/SnO_2_ photocatalyst was decomposed into three peaks. The binding energy around 529.63 eV corresponded to the lattice oxygen of the In-O-In bond, the binding energy around 530.05 eV corresponded to the lattice oxygen of the Sn-O-Sn bond, and the peak at 532.20 eV should be from the oxygen of the -OH group at the composite surface [21]. All the binding energies were negatively shifted relative to those of pure SnO_2_ and In_2_O_3_. The XPS analysis implied the coexistence of SnO_2_ and In_2_O_3_ in the 1.0 wt.% In_2_O_3_/SnO_2_ photocatalyst.

**Figure 5:**
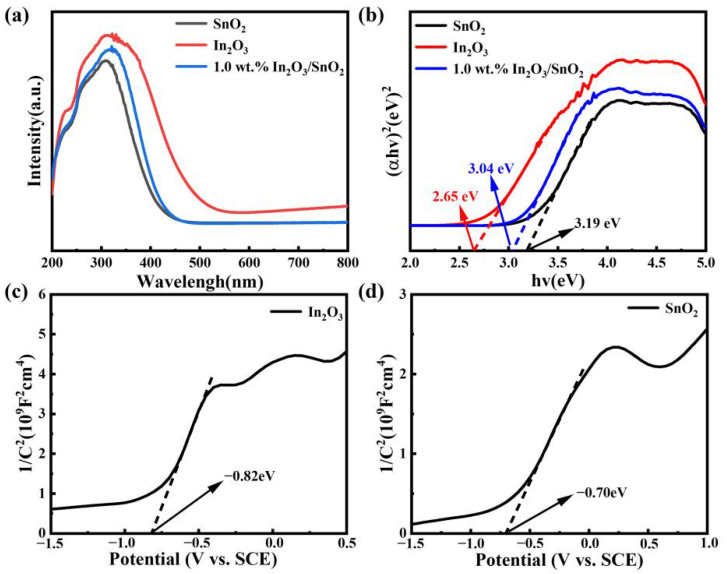
The optical performance of catalytic: (**a**) UV–Vis DRS; (**b**) The bandgap; The Mott–Schottky plot; (**c**) In_2_O_3_; and (**d**) SnO_2_.

The optical absorption characteristics and bandgaps of In_2_O_3_, SnO_2_ and the 1.0 wt.% In_2_O_3_/SnO_2_ were studied by UV–Vis DRS. As shown in Figure 5a, the absorption edges of In_2_O_3_, SnO_2_ and the 1.0 wt.% In_2_O_3_/SnO_2_ were located at about 550, 459 and 455 nm, respectively. Compared with pure SnO_2_, the absorption edge of the 1.0 wt.% In_2_O_3_/SnO_2_ showed a slight red shift, which indicated that the composite photocatalyst had the most reliable absorption capacity. The absorbing edge was calculated using the usual Tauc’s diagram [24]:

(1) $${\left(\alpha hv\right)}^{n}=A\left(hv-E_{g}\right)$$

where *α* is the absorption coefficient, *hυ* is the energy of the incident photon, *E_g_* is the optical bandgap energy (hereinafter referred to as the bandgap energy), A is a constant, and *n* depends on the type of electronic transition. Here, In_2_O_3_ and SnO_2_ were both direct bandgap semiconductors, and their *n* values were both taken as 2.

As shown in Figure 5b, the *E_g_* obtained by In_2_O_3_ and SnO_2_ were 2.65 eV and 3.19 eV, respectively, and the band energy of the 1.0 wt.% In_2_O_3_/SnO_2_ heterojunction was reduced to 3.04 eV, which was due to the strong interaction between In_2_O_3_ and SnO_2_. In order to find out about the separation mechanism of photogenerated cost carriers in the system of degrading pollutants, the CB and VB potentials of In_2_O_3_ and SnO_2_ were preliminarily calculated by Equation (2):

(2) $$E_{CB}=E_{VB}-E_{g}$$

ECB and EVB are the conduction band and valence band potential, respectively. E_g_ is the bandgap of the semiconductor material. As shown in Figure 5c,d, the positive slope of the Mott–Schottky curve confirmed that In_2_O_3_ and SnO_2_ were N-type semiconductors. Compared with saturated Ag/AgCl electrodes, the flat-band potentials of In_2_O_3_ and SnO_2_ were −0.82 and −0.70 eV. The flat-band potentials transformed to NHE were −0.63 eV and −0.51 eV. Meanwhile, the CB position of the N-type semiconductor was 0.1~0.3 eV greater than the flat-band potential [25]. Therefore, the CB potentials of In_2_O_3_ and SnO_2_ were speculated to be −0.93 and −0.81 eV. Combining the CB and bandgap, the VB potentials of the In_2_O_3_ and SnO_2_ were obtained using Equation (2). Therefore, the VB positions of In_2_O_3_ and SnO_2_ were +1.72 eV and +2.38 eV, respectively.

### 3.2. Photocatalytic Performance

**Figure 6:**
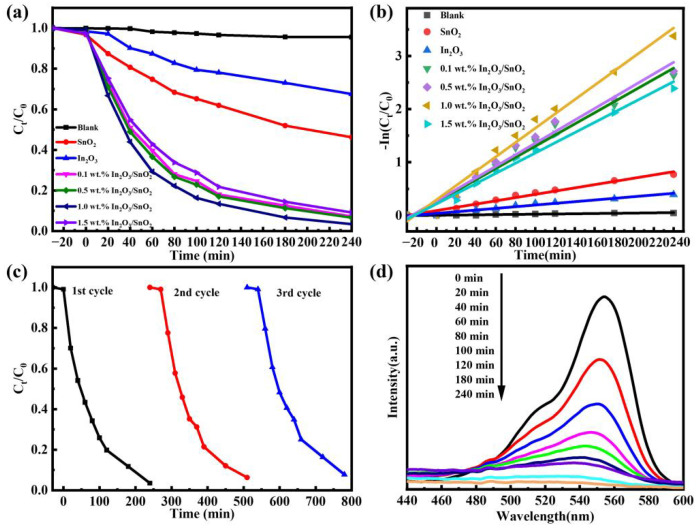
Photo-oxidation activity: (**a**) RhB variation curves; (**b**) degradation kinetics; (**c**) stability test; and (**d**) temporal evolution of UV–Vis spectra during decolorization of RhB.

The degradation of RhB was investigated using the synthesized SnO_2_ samples and In_2_O_3_/SnO_2_ composites under UV light. Figure 6a shows the degradation of RhB. It was observed that the synthesis of the In_2_O_3_/SnO_2_ composites had a synergistic effect on the decomposition of RhB. In total, 54% of RhB was degraded by SnO_2_ after 240 min of UV-light irradiation. The composite of a small quantity of In_2_O_3_ and SnO_2_ considerably improved the effectivity of the photocatalytic degradation of RhB. The degradation rate of the 1.0 wt.% In_2_O_3_/SnO_2_ composite photocatalyst reached about 97% after 240 min, and reached 87% after 120 min of UV-lamp irradiation.

As shown in Figure 6b, the photodegradation kinetics of the synthesized sample in terms of RhB were investigated. Table 1 shows the photodegradation kinetic constant ln(C_0_/C) = ĸ · t, the fitting equation, the rate constant ĸ and the correlation coefficient R^2^. The reaction rate constant of SnO_2_ was 0.00305 min^−1^. The reaction rate constant of the 1.0 wt.% In_2_O_3_/SnO_2_ was five and nine times that of SnO_2_ and In_2_O_3_, respectively. This result also indicated that In_2_O_3_ facilitates the increase in photoactivity.

We studied the stability and reproducibility of the 1.0 wt.% In_2_O_3_/SnO_2_ photocatalyst. Under the identical experimental conditions, two additional cycles of degradation experiments were performed on RhB. Figure 6c indicates that the degradation rate did not change much after three cycles of the photocatalytic process. The synthesized 1.0 wt.% In_2_O_3_/SnO_2_ photocatalyst had good consistency and reproducibility.

The catalytic degradation process of RhB by the samples was studied. As shown in Figure 6d, the UV–Vis absorption spectrum images of the 1.0 wt.% In_2_O_3_/SnO_2_ for RhB dye degradation at unique times were investigated. During photodegradation, the depth of the attribute height of RhB at 554 nm decreased drastically between 0 min and 240 min of light radiation. The maximum absorption peak was shifted from 554 nm to 526 nm. These changes may have been caused by the generation of intermediates in the reaction mixture as well as the n-demethylation and conjugated structure damage of RhB during photodegradation [26]. The disruption of the conjugated chromophore structure of RhB caused a rapid decrease in RhB uptake, indicating that RhB could be degraded to small molecules such as CO_2_ and H_2_O [19]. The above results indicated that the process of n-demethylation and the destruction of the conjugated chromophore structure were synchronized for the duration of the photocatalytic reaction [27].

### 3.3. Catalytic Mechanism

**Figure 7:**
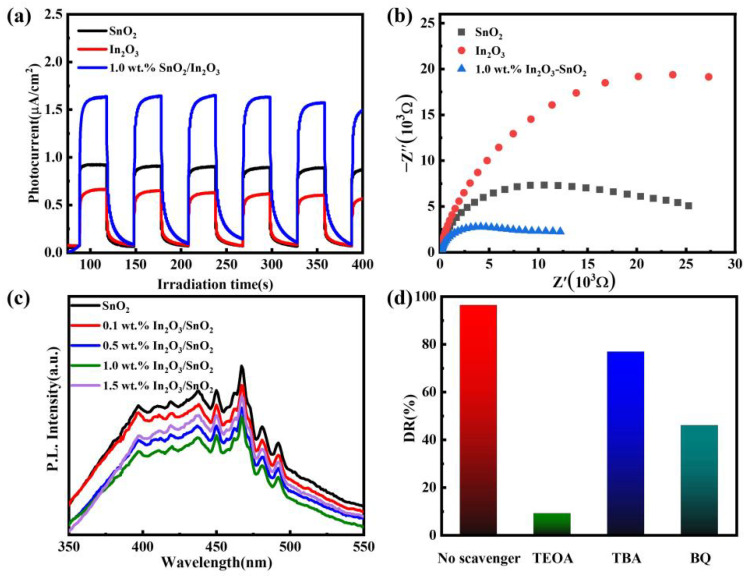
Photoelectrochemical characterization: (**a**) photocurrent of the samples; (**b**) EIS of the samples; (**c**) PL spectra of different samples; and (**d**) degradation of RhB with the addition of BQ, EDTA, TBA.

The electron–hole separation efficiency during photocatalytic degradation was investigated using the transient photocurrent response. As shown in Figure 7a, the photocurrent of the 1.0 wt.% In_2_O_3_/SnO_2_ was higher than that of SnO_2_ and In_2_O_3_. The photocurrent density of the 1.0 wt.% In_2_O_3_/SnO_2_ (1.92 μA·cm^−2^) was 1.5 and 2.3 times that of pure SnO_2_ (1.32 μA·cm^−2^) and In_2_O_3_ (0.84 μA·cm^−2^), respectively. The elevated photocurrent signified that the carriers generated in the 1.0 wt.% In_2_O_3_/SnO_2_ could be separated more efficiently than those in pure SnO_2_ and In_2_O_3_. In general, the electron–hole transport effectivity was positively related to the photocurrent depth [28]. Therefore, in order to acquire higher photocatalytic activity, the 1.0 wt.% In_2_O_3_/SnO_2_ generated a greater number of electron–hole pairs to participate in the photodegradation process. As shown in Figure 7b, the EIS Nyquist plot of the samples was investigated. Compared with the pure SnO_2_, the 1.0 wt.% In_2_O_3_/SnO_2_ composite photocatalyst had the smallest arc radius in the EIS Nyquist diagram. The results indicated that In_2_O_3_/SnO_2_ composite photocatalyst could effectively increase the electron–hole separation rate [29].

Meanwhile, in order to further confirm of the photoactivity of the 1.0 wt.% In_2_O_3_/SnO_2_, photoluminescence (PL) spectroscopy tests were performed. It is well known that PL emission spectroscopy has been widely used to study the capture, transfer, and separation efficiencies of photogenerated charges in semiconductor materials. The lower the PL emission intensity, the lower the recombination rate of photogenerated electron–hole pairs [30]. Figure 7c shows the PL spectra of SnO_2_, 0.1, 0.5, 1.0 and 1.5 wt.% In_2_O_3_/SnO_2_. It can be seen that the luminescence intensity of the composite photocatalysts was lower than that of pure SnO_2_. With the increase in the In_2_O_3_ doping amount, the luminescence intensity first decreased and then increased, and reached the minimum value at 1.0 wt.% In_2_O_3_/SnO_2_. This means that the recombination of electron–hole pairs was hindered and the photocatalytic activity was enhanced [31].

To explore the degradation of RhB by a range of active species, trapping experiments were carried out. The influence of the main substances exposed to the 1.0 wt.% In_2_O_3_/SnO_2_ on the degradation of RhB was studied. As shown in Figure 7d, benzoquinone (BQ) was used to remove •O_2_^−^, triethanolamine (TEOA) to remove h^+^, and tert-butanol (TBA) to remove •OH. After the addition of BQ and TEOA, the effectivity of the degradation of RhB was significantly reduced. The photocatalytic activity of TBA was also decreased, but the decrease was smaller than that of the others. Therefore, •O_2_^−^ and h^+^ played the leading role in RhB degradation, while •OH played an auxiliary role.

**Figure 8:**
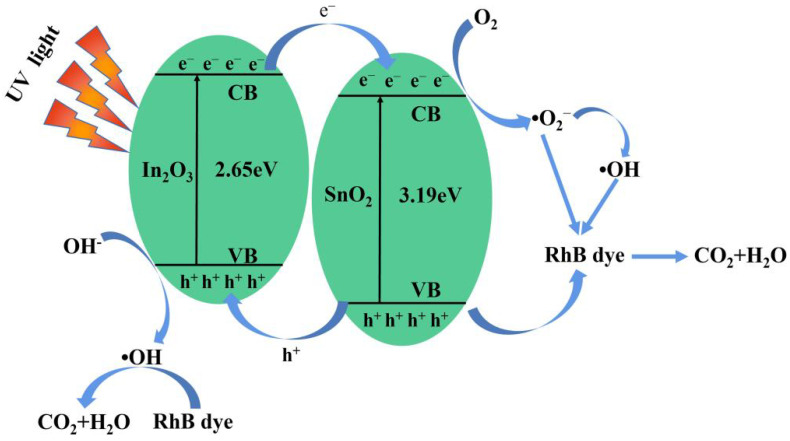
Schematic illustration of the separation and migration mechanism of photogenerated electron–hole pairs of In_2_O_3_/SnO_2_ composites.

According to the above evaluation results, the feasible photocatalytic mechanism of the composite In_2_O_3_/SnO_2_ photocatalyst material was proposed. As shown in Figure 8, the energy band positions of In_2_O_3_ and SnO_2_ were well matched, which can effectively separate photogenerated electrons and holes. In_2_O_3_ and SnO_2_ can be excited by ultraviolet light. A hole (h^+^) was generated in VB, and an electron (e^−^) was generated in CB. The high carrier-recombination rate of pure SnO_2_ led to the poor photocatalytic performance. The synergistic effect of the nanocomposites reduced the composite defects that generate charge, which in turn improved the photocatalytic activity of SnO_2_.

The semiconductor components generate the electron–holes pairs, which is initiated by the absorption of photons with energy equal to or higher than the bandgap (Equations (3) and (4)). A part of the h^+^ of SnO_2_ was transferred to the VB of In_2_O_3_ (Equation (5)), and a part of the e^−^ of In_2_O_3_ was transferred to the CB of SnO_2_ (Equation (6)). Some h^+^ on the surface of SnO_2_ remained in the VB, oxidizing pollutants. The e^−^ reacts with oxygen molecules to form the superoxide anion (•O_2_^−^) (Equation (7)). At the same time, the photocatalytic degradation of RhB in the VB may be due to a direct reaction with holes instead of •OH (Equations (8)–(10)). The viable response steps were summarized as follows [19,27].

(3) $$SnO_{2}+hv\to e^{-}(SnO_{2})+h^{+}(SnO_{2})$$

(4) $$In_{2}O_{3}+hv\to e^{-}(In_{2}O_{3})+h^{+}(In_{2}O_{3})$$

(5) $$h^{+}(SnO_{2})\to h^{+}(In_{2}O_{3})$$

(6) $$e^{-}(In_{2}O_{3})\to e^{-}(SnO_{2})$$

(7) $$e^{-}(SnO_{2})+O_{2}\to •O_{2}^{-}$$

(8) $$RhB+•O_{2}^{-}\to CO_{2}+H_{2}O$$

(9) $$RhB+h^{+}\to CO_{2}+H_{2}O$$

(10) $$RhB+•OH\to CO_{2}+H_{2}O$$

Table 2 shows the photocatalytic activity of the 1.0 wt.% In_2_O_3_/SnO_2_ nanocomposites for the degradation of RhB compared with several recently reported papers for the photocatalytic degradation of organic dyes. The 1.0 wt.% In_2_O_3_/SnO_2_ exhibited both the Fenton effect and photocatalytic activity under UV-light irradiation, verifying that it was an efficient photocatalyst. The reaction conditions such as the concentration of the dye and the content of the catalyst will have a great influence on the performance of the photocatalyst.

## 4. Conclusions

In this study, micron-sized spherical SnO_2_ and flower In_2_O_3_ structures were prepared by a simple hydrothermal method. In_2_O_3_-doped SnO_2_ (In_2_O_3_/SnO_2_) samples with different percentages of In_2_O_3_ contents were prepared by a “two-step method”, and their catalytic performance in RhB degradation was studied. The results showed that the photocurrent density of the 1.0 wt.% In_2_O_3_/SnO_2_ (1.92 μA·cm^−2^) was about 1.5 and 2.3 times that of pure SnO_2_(1.32 μA·cm^−2^) and In_2_O_3_(0.84 μA·cm^−2^), respectively. Compared with pure SnO_2_, the 1.0 wt.% In_2_O_3_/SnO_2_ composite photocatalyst had the smallest EIS Nyquist graph arc radius, which indicated that the In_2_O_3_/SnO_2_ composite photocatalyst could effectively increase the electron–hole separation rate. When the optimal loading capacity of In_2_O_3_ was 1.0 wt.%, the rate constant ĸ value of In_2_O_3_ was five and nine times that of SnO_2_ and In_2_O_3_, respectively. After 240 min irradiation, the photocatalytic performance of In_2_O_3_ was improved from 54% to 97%, and the degradation rate reached 87% after 120 min. These results indicated that the photocatalytic performance of the In_2_O_3_/SnO_2_ composite in RhB degradation was enhanced. A small quantity of In_2_O_3_ modified SnO_2_ to synthesize the composite In_2_O_3_/SnO_2_ materials, which accelerated the photocatalytic efficiency of pure SnO_2_ and suppressed the electron–hole recombination efficiency. Therefore, the In_2_O_3_/SnO_2_ composite was an effective method to improve the photocatalytic activity of SnO_2_.

## Figures and Tables

**Table 1:** The fitted equation, the rate constant ĸ and correlation coefficient (R^2^) of as-prepared samples.

Samples	Fitted Equation	ĸ (min^−1^)	Correlation Coefficient (R^2^)
Blank	ln(C_0_/C) = 0.0002056 t	0.0002056	0.91181
SnO_2_	ln(C_0_/C) = 0.00305 t	0.00305	0.98214
In_2_O_3_	ln(C_0_/C) = 0.00158 t	0.00158	0.96773
0.1 wt.% In_2_O_3_/SnO_2_	ln(C_0_/C) = 0.01049 t	0.01049	0.97336
0.5 wt.% In_2_O_3_/SnO_2_	ln(C_0_/C) = 0.01087 t	0.01087	0.9719
1.0 wt.% In_2_O_3_/SnO_2_	ln(C_0_/C) = 0.0002056 t	0.01347	0.98141
1.5 wt.% In_2_O_3_/SnO_2_	ln(C_0_/C) = 0.0002056 t	0.00958	0.98119

**Table 2:** Comparison of photocatalytic efficiencies of various SnO_2_-based photocatalysts for degradation of organic pollutants.

Catalyst	Pollutant Concentration	Catalyst Dosage (mg)	Light Source	Irradiation Time (min)	Activity (%)	Reference
SnO_2_	^a^ RhB (10 mg/L)	45 mg	high-pressure Hg lamp (175 w)	270 min	~92%	[32]
Fe/SnO_2_	RhB (10 mg/L)	25 mg	UV light (250 w)	120 min	~55%	[33]
Bi_2_O_3_/In_2_O_3_	RhB (10 mg/L)	10 mg	Hg lamp (175 w)	240 min	~92%	[34]
ZnO/SnO_2_	RhB (2 × 10^−6^ M)	50 mg	two 6 W UV tube lamps	120 min	~80%	[35]
1.0 wt.% In_2_O_3_/SnO_2_	RhB (10 mg/L)	50 mg	high-pressure Hg lamp (200 w)	120 min 180 min 240 min	~87% ~92% ~97%	This work

^a^ RhB = Rhodamine B.

## Data Availability

Data presented in this study are available by requesting from the corresponding author.
